# Shellfish as a Source of Bioactive Compounds and Extracts: A Comprehensive Review of Their Anticancer and Antimicrobial Properties

**DOI:** 10.3390/md24020074

**Published:** 2026-02-11

**Authors:** N. M. Liyanage, Li Yiqiao, K. K. Asanka Sanjeewa, Kyung Yuk Ko, D. P. Nagahawatta, You-Jin Jeon

**Affiliations:** 1 Department of Medicine, Faculty of Medicine and Dentistry, University of Alberta, Edmonton, AL T6G 2B7, Canada; 2 Department of Marine Life Sciences, Jeju National University, Jeju 690-756, Republic of Korea; 3 Department of Biosystems Technology, Faculty of Technology, University of Sri Jayewardenepura, Pitipana, Homagama 10206, Sri Lanka; asankasanjeewa@sjp.ac.lk

**Keywords:** shellfish, anticancer, antimicrobial, peptides, fatty acids, functional foods, polysaccharides

## Abstract

Shellfish are a diverse group of marine animals that play a significant role, as a high proportion of the world’s seafood is produced by shellfish. In general, shellfish contain higher amounts of nutrients that benefit consumer health. In recent years, research has focused on the potential health benefits of consuming shellfish, including their anti-cancer and anti-microbial properties. Studies have shown that certain types of shellfish contain bioactive compounds that can inhibit growth and proliferation as well as induce apoptosis in cancer cells both in vitro and in vivo. In addition, shellfish also possess anti-microbial properties which arise from their proteins, peptides, fatty acids, and polysaccharides, which can disrupt the bacterial cell membrane, inhibit bacterial cell division, and interfere with cellular processes. These make them beneficial in preventing and treating infectious diseases. This review explored the findings related to the potential of shellfish bioactive compounds against cancer and microbial infections. Furthermore, this analysis demonstrates unequivocally that shellfish have vast potential for producing functional foods and that the bioactive compounds have the potential to be used in pharmaceutical applications.

## 1. Introduction

The oceans are an essential natural resource on Earth, as they provide sustenance, mainly in the form of fish and shellfish. Shellfish, a significant component of global aquatic food consumption, consists primarily of crustaceans and mollusks. Crustaceans have segmented bodies with hard shells made of chitin, whereas mollusks possess soft bodies divided into the foot and visceral sections. Mollusks are further divided into bivalves, cephalopods, and gastropods. The commercially important bivalves are mussels, oysters, clams, and scallops, while cephalopods include squid, cuttlefish, and octopus. Among other things, the gastropod family includes abalone, sea snails, cockles, and whelks. More than 1000 species of crustaceans, 50,000 types of mollusks, and 13,000 species of fish are estimated to live in the ocean [1]. Shellfish are a vital source of food and income for many coastal communities, and their consumption has been associated with numerous health benefits due to their high levels of protein, omega-3 fatty acids, and other essential nutrients. The diverse ecosystem that marine shellfish occupy is subject to harsh environmental conditions. They, therefore, create a variety of powerful active ingredients that have important biological activities. These compounds have gained the scientific community’s interest in their nutritional value and prospective significance in the nutraceuticals and functional foods industries.

In search of bioactive metabolites, the scientific community has paid significant attention to shellfish, extracting around 1145 natural compounds during the past 30 years [2]. Numerous researchers have studied the biological activities of shellfish parts throughout the years, and several dietary supplements containing shellfish extracts have been released on the market. For instance, an anti-inflammatory and anti-arthritic supplement called Lyprinol^®^, which includes a lipid extract from the green-lipped mussel *Perna canaliculus*, is available globally. Recently, there have been several studies conducted on the anti-inflammatory, antioxidant, and antihypertensive potential of shellfish [3,4,5,6,7]. However, the reported anticancer and antimicrobial properties of shellfish-derived compounds are scarce.

Marine shellfish are a rich source of bioactive compounds that have been found to possess significant anticancer and antimicrobial properties. The potential health benefits of these compounds have been a subject of interest for researchers and have created opportunities to develop novel therapeutic approaches focused on therapeutic targets. Anticancer and antimicrobial agents are in high demand due to the increasing incidence of cancer and antibiotic-resistant bacterial infections. Marine shellfish-derived compounds offer a promising source of natural products that could potentially be developed into new therapeutic agents. Therefore, this review is intended to compile information on marine shellfish-derived compounds related to health complications such as cancer and microbial diseases. Additionally, our work will serve as a foundation for future investigations into nutraceuticals, functional foods, and the dietetic assessment of marine shellfish.

## 2. Methodology

This review aims to analyze the anticancer and antimicrobial activities of shellfish-derived compounds and extracts. A comprehensive literature search was conducted across electronic databases, including PubMed, Scopus, and Web of Science, covering studies published between January 2000 and December 2024. This extended time frame was intentionally selected due to the limited number of studies specifically investigating shellfish-derived bioactives, particularly in the context of anticancer and antimicrobial activities.

The search strategy employed combinations of keywords such as “shellfish”, “marine mollusks”, “crustaceans”, “bioactive compounds”, “peptides”, “lipids”, “polysaccharides”, “anticancer”, “antimicrobial”, “bacteria”, “fungi”, and “virus”. In addition, the reference lists of relevant articles were manually screened to identify further pertinent studies. Studies were included if they examined anticancer or antimicrobial activities of shellfish-derived extracts, fractions, or defined compounds using in vitro or in vivo experimental models. Studies were excluded if they lacked appropriate controls, did not involve cancer or microbial targets, or were not published in English. Reviews, conference abstracts, and studies focusing solely on non-shellfish marine organisms were also excluded.

From eligible studies, information was extracted on compound type, shellfish source, experimental model (cell line, microorganism, or animal model), tested concentrations, biological outcomes, and key mechanistic insights. Due to the heterogeneity of experimental designs, compound classes, and outcome measures across the literature, data were synthesized using a narrative review approach rather than a systematic meta-analysis. This approach allowed for a comprehensive and contextual evaluation of current evidence and the identification of emerging trends and research gaps in the field.

## 3. Bioactive Compounds from Shellfish

Various health benefits have been reported from shellfish, ranging from anti-viral, anti-inflammatory, and antioxidant to antimicrobial functions. Mollusks have emerged as a rich source of bioactive compounds with significant potential to promote human health and aid in the prevention and treatment of chronic diseases. These marine organisms produce a diverse array of molecules with various biological activities that have attracted considerable scientific interest.

The shellfish-derived proteins/peptides, fatty acids and shell-derived polysaccharides have been investigated and reported for their anticancer and antimicrobial activity compared to other activities.

### 3.1. Proteins and Peptides

Proteins are complex molecules vital for the proper functioning of all living organisms, making up approximately 65–75% of the dry weight in some animal tissues. Marine-derived proteins, particularly from shellfish, have garnered considerable attention for their bioactive and functional properties. Shellfish contain significant amounts of high-quality proteins, ranging from 10–47% (*w*/*w*), making them excellent sources for bioactive protein exploration [8]. Proteins are polymers composed of amino acids and peptides, with shellfish protein content varying from 7 to 23% (*w*/*w*).

Shellfish proteins are categorized into three main types: sarcoplasmic, myofibrillar, and stromal proteins. Sarcoplasmic proteins, such as creatinine kinase, aldolase, and glyceraldehyde-3-phosphate dehydrogenase, are enzymes involved in energy production. Myofibrillar proteins are structural components of fish and shellfish muscles. Additionally, paramyosin, a protein found in invertebrate muscles, is absent in vertebrate myofibrils, with its levels varying significantly across species [9]. Protein hydrolysates, along with hemolymph and hemocytes, play essential roles in the innate immune system of invertebrates, preserving immune components like lectins, antimicrobial peptides (AMPs), coagulation factors, and protease inhibitors. These elements modulate the immune response and hold therapeutic potential.

Research on functional peptides that modulate immune responses is rapidly evolving and shows significant promise. Enzymatic hydrolysis of shellfish, which is rich in proteins, results in the formation of bioactive peptides. These shellfish-derived peptides have been documented in various animal studies for their immune-stimulating properties, although there is a notable lack of relevant clinical studies [10]. Peptides extracted from shellfish protein hydrolysates have demonstrated the ability to effectively stimulate immune cell activities. They enhance lymphocyte proliferation, increase the activity of natural killer (NK) cells, and regulate cytokine production. Despite these observed effects, the precise mechanisms underlying these immune responses remain poorly understood [11]. Bioactive peptides normally have 3–20 amino acids, and the action of the peptide depends on its sequence and composition of amino acids [12]. It is reported that peptides having low molecular weight have a higher potential to pass through the intestinal wall and exert biological activities. Recently, increasing attention has been given to the compositional, sequential, and structural characteristics of shellfish bioactive peptides. Beyond their traditional role as nutrients, specific amino acid sequences have been recognized for their significant involvement in various biological functions. These include anticancer, antithrombotic, antihypertensive, antioxidant, opioid agonist or antagonist, immunomodulatory, and antimicrobial activities [13].

### 3.2. Lipids/Fatty Acids

Lipids belonging to the fundamental group of nutrients for humans contribute to the structure of biological membranes and act as an energy source as well as a key signaling molecule [14]. The principal role of lipids in marine organisms consists predominantly of triacylglycerol and wax esters, which are a reserve of fatty acids (FA) that are destined for oxidation to energy production or incorporation into phospholipids. Fatty acids are classified into saturated and monounsaturated fatty acids.

Nowadays, essential FAs are considered to be functional foods and nutraceuticals with many benefits for human health, including the potential to reduce the risk of cardiovascular diseases, cancer, osteoporosis, and diabetes [15]. Omega-3 polyunsaturated fatty acids (PUFAs) are essential fatty acids primarily synthesized by algae and transferred through the marine food chain, making marine animals one of the richest sources of these beneficial fats. Shellfish typically contain PUFAs that range from 1.5% to 10.5% of their total fatty acid composition. Numerous studies have highlighted the positive effects of dietary PUFAs on the immune systems of both humans and animals [16].

Lipids extracted from the liver of Australian lobsters were identified to be useful in the treatment and prevention of various conditions, including coronary heart disease, arthritis, asthma, and cancers, due to the high concentrations of PUFAs and low levels of lead, arsenic, and mercury-like contaminants [17]. A notable example of a commercialized marine-derived product is Lyprinol^®^, a lipid extract derived from the freeze-dried, green-lipped mussel (*P. canaliculus*), which is farmed in New Zealand. Lyprinol^®^ has gained attention for its potent bioactivities and is currently marketed as a supplement to help reduce inflammation associated with conditions such as arthritis [18].

### 3.3. Polysaccharides

Shellfish polysaccharides are one of the most attractive compounds among other functional ingredients. The uniqueness of shellfish-derived polysaccharides is due to the presence of glycosaminoglycans and polysaccharide sulfates, which are the primary metabolites of shellfish [19]. The polysaccharide compositions and content varied greatly in different shellfish species. Shi et al. reported that polysaccharides isolated from *Cipangopaludina chinensis* contain D-glucose with a molecular weight of 91.1 kDa. He further reported that the backbone was composed of (1 → 3) linked α-d-Glc with branches consisting of a single (1 → 3) linked α-d-Glc unit at the C-4 position and terminal α-d-Glc-4-O-SO3 linked to O-3 of (1 → 3) linked α-d-Glc units [20]. A heteropolysaccharide isolated from *Scapharca subcrenata* was found to be composed of multiple monosaccharides, including galacturonic acid, glucose, galactose, mannose, ribose, rhamnose, fucose, xylose, and arabinose. These components were present in a molar ratio of 1.00:5.40:9.04:3.10:1.59:4.01:2.10:2.21:2.28, respectively [21].

The polysaccharides from shellfish have various physicochemical properties like solubility, crystallinity, thermal stability, and emulsification. Shellfish polysaccharides exhibit various biological activities such as hepatoprotection, immunomodulation, anti-inflammatory, and anti-cancer. These biological activities are mainly attributed to their chemical structure [19].

Table 1 summarizes several shellfish-derived bioactive compounds and their bioactivities.

## 4. Anticancer Activities of Bioactive Compounds from Shellfish

Cancer, the dreadful human disease, is a collection of pathologies related to the unrestrained proliferation of cells, having a negative impact on human health care. As reported by the World Health Organization (WHO) in 2019, cancer is the primary or second major cause of death before the age of 70 in 112 of 183 nations, and it ranks third or fourth in another 23 countries [50]. Breast cancer is the most prevalent and deadly kind of cancer among women, whereas lung cancer is the most prevalent and deadly type of cancer among men. Despite the advancements in medical research, immunotherapy, radiotherapy, surgery, and chemotherapy, conventional therapeutic approaches result in significant damage to vital organs surrounding the tumor [51]. Additionally, drug tolerance precludes the efficacy of other therapeutic modalities, rendering the treatment ineffective. All these factors should be considered when developing effective cancer therapies. This encourages researchers to investigate novel anticancer therapeutics that are selectively cytotoxic to cancer cells and safe for normal cells. Natural bioactive substances are reported to have a range of therapeutic potential as anticancer medications. According to reports, the source of around 60% of anticancer medications is natural substances or their derivatives [52,53]. Natural products have been one of the main sources of compounds for drug discovery and have demonstrated potential in the biomedical field due to their bioavailability and great structural diversity, which results in numerous co-occurrences. Among the marine natural sources, shellfish have gained a dramatic increase in interest in recent years due to the availability of numerous metabolites that suppress the development of human tumor cells in vitro, in vivo (in mouse models), and in cancer clinical trials, acting as antitumor medicines.

Bivalve mollusks, including oysters, are consumed by people in all parts of the world. Edible oysters have been consumed by people for at least 700 years. Oysters contain a lot of protein, and oyster peptides have been reported to possess anticancer activity. A study conducted by Wang and colleagues (2010) reported the isolation of oligopeptide-enriched hydrolysate from oyster (*Crassostrea gigas*) using protease enzyme [54]. The protease was prepared from *Bacillus* sp. SM98011 and the extraction was carried out at pH 7.5 and 50 °C. In the investigation on *C. gigas* protein hydrolysate on antitumor and immune-stimulating activity in BALB/c mice, they identified that the growth of transplantable sarcoma S180 was inhibited by the protein hydrolysate. According to the study conducted by Kim and colleagues (2010), *C. gigas* possesses anticancer activity in PC3 cells [55]. The lipid extracts of *C. gigas* were prepared using organic solvents, and the extracts were evaluated. Hexane extracts were proven to possess superior anticancer activity against human prostate tumor growth through apoptosis in cancer cells.

Another group of researchers has evaluated the anticancer activity of a peptide isolated from the protease-assisted enzymatic hydrolysate of *Saccostrea cucullata* [56]. According to their findings, the peptide (Leu-Ala-Asn-Lys) isolated by gel filtration chromatography of oyster hydrolysate showed significant inhibition of human colon carcinoma (HT29) cell viability and increased apoptosis when treated. Moreover, they identified that the peptide contained significant dose and time-dependent anticancer effects on cancer cells. A study conducted on *R. philippinarum* reported its anticancer activity against prostate cancer cell proliferation. In the study, eight proteases were applied for the enzymatic hydrolysis. Among the hydrolysis, α-Chymotrypsin hydrolysate showed superior activity, and a novel anticancer peptide was purified. Further, the authors reported that the peptide Ala-Val-Leu-Val-Asp-Lys-Gln-Cys-Pro-Asp, effectively induced apoptosis in prostate, breast, and lung cancer cells [57]. Colorectal carcinoma (CRC) is one of the major causes of cancer-related incidence and deaths. A study conducted on *Arca inflata* has reported that a peptide, P6, isolated from the bivalve mollusk has the potential to markedly inhibit cell proliferation and colony formation, and induced apoptosis in CRC cells. Through mechanistic studies they have identified that P6 induced colon cancer cell apoptosis through the activation of the p38-MAPK signaling pathway. Moreover, it was demonstrated that P6 exhibited antitumor effects in a tumor xenograft model, and induced cell cycle arrest in CRC cells in a concentration-dependent mode [58].

Apart from the peptides from oyster, carbohydrates extracted were also proven to fight against cancer. *C. gigas* was subjected to a simple aqueous two-phase system, and oyster polysaccharides were purified. The polysaccharides were tested against Hepatoma Gell line 2 (HepG2) cells, and it was reported that the polysaccharides stimulate phagocytic activity and IL-2 production in cells, resulting in excellent anti-tumour activity [59].

Most blue mussels (*Mytilus edulis*) are cultivated in Canada, the United States, Europe, and Africa, and it is generally known that they have great nutritional potential as a source of protein, vitamin C, iron, zinc, and -3 PUFAs. Beaulieu et al. (2013) [60] used a commercial *Bacillus* protease complex with broad sensitivity to hydrophobic amino acids to extract hydrolysates of entire blue mussels. Following molecular mass-based fractionation, four immortalized cell lines, A549 (type II pulmonary epithelial cells), HCT15 (colon carcinoma cells), BT549 (breast carcinoma cells), and PC3 were used to investigate the various fractions for anti-proliferative activities. Only the 50 kDa hydrolysate fraction, which is rich in peptides, was shown to have anti-proliferative effects on all the cell lines that were examined, with strong activity towards the PC3 and A546 cells. *M. coruscus* cultivated in Korea was investigated for its anticancer potential by Kim et al. (2011) [61]. The lipid extracts of the mussels were reported to have high anticancer activity against human prostate, breast, lung, and liver cancer cells. The extract was then further separated and purified by thin layer chromatography, and they have identified the major fatty acid in the extract were eiocosadienoic acid (EA, C20:2), eicosapentaenoic acid (EPA, C20:5), and docosahexaenoic acid (DHA, C22:6). Furthermore, a separate study was conducted on *M. coruscus* enzymatic hydrolysate in 2012 has reported the anticancer activity of anticancer peptide isolated from the mussel species. In the study, eight proteases were examined for their anticancer effect on the prostate cancer cell line. Among the protein hydrolysates, pepsin hydrolysate showed a clear and superior cytotoxic effect, and it was purified to obtain the anticancer peptide “Ala-Phe-Asn_Ile-His-Asn-Arg-Asn-Leu-Leu”. They observed that the peptide induced cell death in prostate cancer cells [62].

Another study conducted on two ceramide derivatives isolated from mussel *Bathymodiolus thermophilus* indicated that the derivatives induce apoptosis in cancer cells. This was assessed via Apopscreen cell-based screening, and the structural characterization was done using a combination of Nuclear Magnetic Resonance (NMR) spectroscopy, mass spectrometry, and chemical degradation [63]. Kovalchuk and colleagues implemented a study that a new lectin family with a β-trefoil fold is represented by the GalNAc/Gal-specific lectin from the sea mussel *Crenomytilus grayanus* (CGL). They further stated that these lectins demonstrate anticancer activity against breast cancer cells [64]. Further, they explained that the isolated lectin recognizes globotriose on the surface of breast cancer cells and binds to the active site, resulting in inhibition of cell signaling mechanisms. The sea mollusk *C. grayanus* from Japan was examined by Liao and fellow researchers due to its popularity as a source of bioactive compounds [65]. They have found out that the crystal structures of lectin extracted from *C. grayanum* attached to galactose, galactosamine, and globotriose, and it was shown that each may bind three ligands via a carbohydrate-binding motif, including a substantial hydrogen bond network mediated by histidine and water. They further observed that the lectin is identified on the surface of breast cancer cells, which results in cell death.

Clams are bivalve mollusks that exist in both marine and freshwater. By using ammonium sulphate fractionation, ion exchange, and hydrophobic interaction chromatography, Ning et al. (2019) [66] isolated and purified the 40 kDa protein from the coelomic fluid of Meretrix and assessed its in vitro anticancer efficacy against several cancer cell lines. Human hepatoma BEL-7402 cell growth in vitro was strongly inhibited by the pure protein extract, with an IC50 of 52.2 g/mL. Although no effect was seen on the growth of benign cells like murine fibroblasts NIH3T3 and human breast epithelial MCF-10A cells, even at a concentration as high as 300 g/mL. This protein extract also demonstrated cytotoxicity against human breast cancer MCF-7 and human colon cancer HCT116 cells. They further reported that the protein fraction disrupts the cancer cell membrane, leading to cell death. Wang et al. (2009) [11] used ammonium sulphate fractionation, ion exchange, gel filtration, and reverse phase chromatography to purify a new polypeptide known as Mere15. It was discovered that Mere15 exhibited cytotoxicity against several human cancer cell lines. Additionally, it was shown that Mere15 greatly inhibited the development of the human lung cancer A549 xenograft in nude mice. They further stated that the mechanism of the anticancer activity is via the G2/M phase arrest followed by cell apoptosis. And, at higher concentrations, it inhibits cell adhesion, migration, and invasion. The effects of Mere15 on the in vitro growth of human K562 chronic myelogenous leukaemia K562 cells were also studied by Liu et al. (2021) [37]. They discovered that Mere15 had an IC50 value of 38.2 g/mL and inhibited the development of K562 cells. Additionally, they showed that the apoptosis was induced concentration-dependently in K562 cells, leading to an excess of reactive oxygen species (ROS) and a loss of mitochondrial membrane potential. Mere15 may thus be regarded as a broad-spectrum anticancer polypeptide that demonstrates cytotoxicity against both chronic myelogenous leukaemia cells and solid cancer cells. Popular shellfish called black clams (*Chione fluctifraga*) are a significant source of income for the people living in the Gulf of California. According to the size of the proteins, Garcia-Morales et al. (2016) [67] produced the protein extract of the entire black clam and separated it into high- and low-molecular weight fractions using gel filtration chromatography. The MDA-MB-231 breast cancer cell line, the Henrietta Lacks (HeLa) cervical cancer cell line, and the ARPE-19 human retinal pigment epithelial cell line were used as test subjects for these fractions’ in vitro antiproliferative abilities. It was discovered that these fractions prevented the MDA-MB-231 and HeLa cells from proliferating in vitro.

Blood clams (*Tegillarca granosa*) are widely distributed in the Indo-Pacific region, and Chi and colleagues (2015) have investigated its anticancer potential in PC-3, DU-145, H-1299, and HeLa cell lines [68]. They have tested two peptides isolated from the Neutrase protein hydrolysate of clam species and reported that the peptide Trp-Pro-Pro significantly altered the morphology of PC-3 cells. Moreover, the peptide was proven to induce apoptosis in PC-3 cells when treated. Likewise, *Donax variabilis*, was another clam species proven to possess anticancer activity. As reported by Sahayanathan et al. (2020) in their study, a polysaccharide was isolated from the whole tissue of the clam species and treated on human lung cancer cells (A549 cells) [69]. A significant inhibitory effect on A549 cells was observed with nuclear, cellular, and apoptotic morphological changes and DNA damage. The polysaccharide was further explained to exhibit cell cycle arrest via the downregulation of cell cycle regulators such as Cyclin A and cdk2.

Many nations in East and Southeast Asia have considerable abalone farming and use shellfish to be a delicacy. According to Uchida et al. (1987) [70], the glycoprotein fraction of the liquid obtained from heating abalone (*Haliotis discus hannai*) significantly slowed the growth of tumors in ICR mice or BALB/c mice that had been subcutaneously injected with either allogeneic sarcoma 180 or syngeneic Meth-A fibrosarcoma. Additionally, the fraction was discovered to stimulate peritoneal and alveolar macrophages’ in vitro cytostatic activity. These findings showed that the host-mediated response was stimulated rather than a direct toxic impact as the cause of the anticancer action. Using 4T1 murine mammary carcinoma cells, Lee et al. (2010) [71] assessed the anticancer efficacy of abalone visceral extract on a breast cancer model. They discovered that oral treatment of abalone visceral extract dramatically slowed the development of the main tumor as well as metastatic lesions by lowering the levels of Cyclooxygenase-2 (Cox-2), Epidermal Growth Factor (EGF), Vascular Endothelial Growth Factor (VEGF), and fibroblast growth factor (FGF). Additionally, it was shown that this extract enhanced CD8+ T cell proliferation and cytolytic activity.

Three polysaccharides, AAP, AVAP I, and AVAP II, were extracted from abalone (*H. discus hannai*) extract and tested for their impact on the growth of a human liver cancer cell line in a recent research by Wang et al. (2014) [72]. From the study, they concluded that the oligomer of AVAP1 suppresses the proliferation of human cancer cells, leading to cancer protective activity. The *H. discus hannai* abalone species was used in another study to evaluate anti-metastasis and anti-pro-angiogenic factors and mechanisms in human fibrosarcoma (HT1080) and human umbilical vein endothelial cells (HUVECs) [73]. The authors have extracted peptides from the by-products of the abalone species, and the findings showed that BABP therapy greatly reduces HT1080 cell migration and HUVEC invasion. By preventing mitogen-activated protein kinases (MAPKs), NF-B signaling, and hypoxia, BABP reduces the production and activity of matrix metalloproteinase (MMP) that is triggered by the hormone phorbol 12-myristate 13-acetate (PMA)-induced vascular endothelial growth factor (VEGF) release and build-up of the hypoxia-inducible factor (HIF)-1 signaling pathway. Vasculogenic mimicry (VM), which is created by tumor cells, is crucial to the development of the tumor because it feeds the cells and removes metabolites. As a result, VM inhibition is essential for the therapeutic management of malignancies. A study conducted on *H. discus hannai* showed that the isolated novel peptide contains potent anti-tumor activity against HT1080 cell migration and invasion during cancer progression [74]. The study reported that the peptide effectively inhibited the MMPs through MAPK and NF-κB pathway obstruction, which led to suppression of metastasis of tumor cells.

Shellfish polysaccharides have shown anti-proliferative activity in various cancer cell lines. As reported by Jiang et al., polysaccharide fraction isolated from *Cyclina sinensis* possesses strong anti-cancer activity against human gastric cancer. The isolated polysaccharide was proven to have strong scavenging activity on superoxide radicals and inhibit the growth of human gastric cancer cells such as BGC-823 [75]. A novel polysaccharide (CFPS-1) isolated from *Corbicula fluminea* was proven to have anticancer potential against human breast cancer cells MCF-7 and MDA-MB-231. It includes S-phase cell cycle arrest and apoptosis in MCF-7 by modulating key proteins such as p53, p21, Bax, and cleaved caspase-7 while suppressing Cdk4, cyclin D, and caspase-7. Further, the isolated CFPS-1 induces apoptosis in MDA-MB-231 cells through sub-G1 phase, driven by a similar regulation of apoptotic proteins [76]. The low molecular weight polysaccharides mainly isolated from oysters have the ability to induce NF-kB p65 nuclear translocation and reverse IFN-γ profile, which stimulates the activity of dendritic cells infiltration into the tumor tissues, resulting in tumor growth suppression [77]. JNY2PW, a highly branched α-D-glucan purified from the traditional marine Chinese medicine *A. inflata* has demonstrated notable antitumor activity both in vitro and in vivo [47]. It effectively reduced tumor cell proliferation and suppressed CXCL5 expression. Additionally, JNY2PW inhibited epithelial–mesenchymal transition (EMT) in both wild-type and CXCL5-overexpressing tumor cells. In tumor-bearing mice, it exerted its antitumor effects by modulating the Akt/mTOR and ERK/GSK3β/β-catenin signaling pathways, while attenuating CXCL5 expression, all without causing significant toxicity.

**Figure 1:**
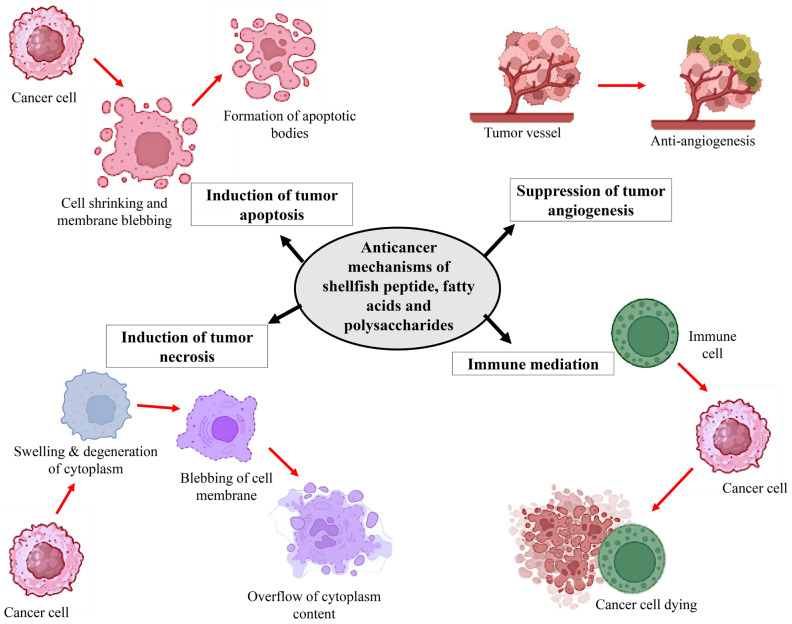
Anticancer mechanisms of shellfish secondary metabolites.

Additionally, the anticancer drugs developed from marine mollusks are summarized in Table 2. Figure 1 summarizes the different anticancer mechanisms of shellfish secondary metabolites.

## 5. Antimicrobial Activities of Secondary Metabolites from Shellfish

Despite the enormous advancements in human medicine, infectious illnesses brought on by bacteria, fungi, and viruses continue to threaten the public’s health. Due to the relative scarcity of medications and the establishment of widespread drug resistance, they have a particularly significant impact in underdeveloped nations. There has been a desire for the creation of novel and potent antimicrobial chemicals because of the ongoing evolution of microbial diseases toward antibiotic resistance. The creation of new antimicrobial chemicals for therapeutic use is one answer to the worldwide challenge of antibiotic resistance. Marine environments have remained essentially undiscovered for their capacity to create pharmaceutical metabolites in comparison to the terrestrial environment, which was the focus of the pharmaceutical industry for more than 50 years. To identify fresh leads for medication candidates, especially from marine shellfish, research has spread from the land to the water during the previous few decades.

Tsankova et al. (2021) have evaluated the antimicrobial potential of different extracts from the Black Sea mussel (*Mytilus galloprovincialis*) against pathogenic bacteria *Staphylococcus aureus*, *Escherichia coli*, and *Klebsiella pneumonia* [95]. Extraction of the mussel was done using several solvents such as ethyl acetate, methanol, glycerol:water, ethanol, acetone, and hot water, and the antimicrobial activity was assessed using the disc diffusion method. According to the results, ethyl acetate extract exhibited a strong antibacterial effect against *E. coli* (13 mm) and *K. pneumoniae* (11 mm) and did not show any activity against *S. aureus*. The growth rate of *S. aureus* (11 mm) and *E. coli* (10 mm) was significantly reduced by the glycerol:water extract, while the growth rate of *K. pneumoniae* was not affected. In another study, the anti-*Bacillus megaterium* activity of three common bivalve mollusks was measured. An oyster, *Crassostrea virginica*, and two mussels, *Geukensia demissa* and *M. edulis*, were used in the study. *Perkinsus marinus* pathogens from *C. virginica* were tested against the plasma samples from these bivalves. As reported, strong anti- *B. megaterium* activity was observed in plasma from *C. virginica* and *M. edulis* but not in *G. demissa*. Furthermore, peptides (10 kDa) were extracted from the plasma samples using gel filtration, and mild antibacterial peptide activity was evaluated in *C. virginica* peptides but not in mussel peptides [1,96].

Bhakata and colleagues have investigated the antibacterial activity of marine Mollusca *Cypraea* sp. and edible oyster *Saccostrea cucullata* against five pathogenic bacterial species, namely *E. coli*, *K. pneumoniae*, *S. aureus*, *Shigella dysenteriae*, and *Enterococcus faecalis*. They used the whole-body water and solvent extracts of the shellfish species and tested the antibacterial activity using the disc diffusion method. According to the results obtained, acetone extract and butanol extract from *S. cucullata* containing fatty acids showed superior activity against the five human pathogenic bacteria [97]. A study has evaluated the antibacterial activity of protein hydrolysate and chitosan prepared from *Sepia kobiensis* cuttlebone and phosphorylated chitosan derived after reaction with orthophosphoric acid against human pathogenic bacteria *Streptococcus pneumoniae*, *S. aureus*, *E. coli*, *Vibrio cholerae*, *V. alginolyticus*, *Vibrio parahaemolyticus*, *Pseudomonas aeruginosa*, *K. pneumoniae*, *Salmonella* sp., and *Proteus vulgaris*. The results indicate that the antibacterial activity was effective against all the bacterial species, and the activity was found to be concentration-dependent. In 100% concentration of chitosan, the highest inhibition zone was observed against *P. vulgaris* (17 mm), while in the same concentration of phosphorylated chitosan, the highest inhibition zone was recorded against *S. aureus* (16 mm) [98]. Against *S. pneumoniae* in chitosan and *P. vulgaris* in phosphorylated chitosan, the lowest inhibition zone of 13 mm and 7 mm, respectively, was demonstrated [98].

Another study has evaluated the antibacterial effect of different extracts from *Macrobrachium nipponense* prawn shells against pathogenic bacteria, *Bacillus subtilis*, *S. aureus*, *K. pneumoniae*, *V. cholerae*, and *E. coli* by using the paper disk diffusion method. Hydroalcoholic, methanol, and acetone extracts of the prawn shells have been tested against the bacteria, and according to the results, all the extracts exhibit antibacterial activity. Further, *B. subtilis* (12.12 ± 0.32 mm), *S. aureus* (12.51 ± 0.14 mm), and *V. cholerae* (12.35 ± 0.27 mm) bacterial strains showed the highest inhibition zone. Moreover, against all the extracts, *S. aureus* showed a significant inhibition zone (*p* < 0.05). This evidence proves the antibacterial effect of prawn extracts [99]. Chandran et al. (2009) have reported the antimicrobial activity of the gill extract of the Asian brown mussel, *Perna viridis*, against pathogenic bacteria and fungi. According to the antibacterial effect results, the highest inhibition zone of 19 mm has been observed against *S. aureus*, while the minimum inhibitory zone has been observed against *Salmonella paratyphi*, which is 11 mm. The gill extraction also exhibited antifungal activity against *Aspergillus flavus* with a 13 mm maximum inhibition zone and for Mucor species with an 11 mm inhibition zone. SDS-PAGE results obtained by the gill extraction samples with antimicrobial activity have shown one clear band in the gel, which is 9.7 kDa of molecular weight [100]. These results indicate the antibiotic potential of *P. viridis*.

A study has evaluated the antifungal activity of chitosan extracted from the exoskeleton of shrimp against *Aspergillus niger* and *Aspergillus oryzea* by using agar tube dilution method. According to the results, at 50 µg/mL of chitosan concentration, both fungi species, *A. niger* and *A. oryzea*, showed a maximum inhibitory zone of 15 mm and 16 mm, respectively. Fluconazole has been used as the positive control, and the inhibition zone of the positive control at a 50 µg/mL concentration has resulted in a 17 mm inhibition zone. These results indicated the strong antifungal activity of chitosan extracted from shrimp exoskeleton compared to the standard drug Fluconazole. Further, in vitro cytotoxic assay performed by using yeast cells has resulted in a decrease in yeast cell viability with increasing chitosan concentration. Moreover, 50 µg/mL chitosan concentration treatment has reduced the yeast cell viability up to 61% [101]. These findings demonstrate the antifungal potential of chitosan extracted from shrimp exoskeletons. It is generally known that lectins (proteins that bind to carbohydrates) actively engage in the defensive mechanisms of both vertebrates and invertebrates, where they play a crucial role in the identification of foreign particles. In a study, the mussel *C. grayanus* was subjected to an in vitro antifungal activity investigation. In the study, lectin was isolated from the mussel species and tested, and the results indicated that the mussel lectin inhibited the spore germination of the fungi and reduced the hyphal growth. The authors further concluded that the lectin from *C. grayanus* is involved in the innate immune response [102].

Xing et al. (2013) reported on the utilization of waste shells from marine shellfish that contain antifungal compounds [103]. According to their study, the heat-treated scallop and oyster shell waste powder slurry was subjected to antifungal activity against *Physalospora piricola* Nose and *Rhizoctonia solani* Kuhn. The antifungal assays were evaluated in vitro by the mycelium growth rate test. The study proved that the scallop and oyster shell powder possesses antifungal activity against both fungi. Further, they reported that oyster shell powder affects the membrane permeability of the fungi and exhibits its antifungal activity. A study conducted by Chan et al. (2021) reported that the tissue extracts prepared using n-hexane from *Magallana bilineata* and *Magallana cuttackensis* oysters contain antifungal compounds [104].

Agar well diffusion assay was implemented in screening bactericidal activity against *E. coli*, *K. pneumoniae*, *Shigella species*, *Salmonella typhi*, *Salmonella paratyphi A*, *Salmonella paratyphi B*, *P. aeruginosa*, *Proteus mirabilis*, and *Enterobacter* species, and fungicidal activity against *Staphylococcus saprophyticus*, *Staphylococcus epidermidis*, *B. subtilis*, *Bacillus cereus*, *Micrococcus luteus*, *Corynebacterium xerosis*, and vancomycin resistant Enterococci i.e., *Enterococcus faecalis*. The results state that *M. bilineata* has more potent antibacterial properties than *M. cuttackensis*. Compared to ciprofloxacin and miconazole, *M. bilineata* could be able to suppress bacterial growth with a low inhibitory concentration (0.75–20 g/mL) and fungal pathogens (0.75–5 g/mL). These results proved the antifungal activity of marine shellfish and their derived compounds.

Novoa et al. (2016) have reported the antiviral activity of nano-encapsulated myticin C (Myt-Tat), a modified peptide derived from the Mediterranean mussel (*M. galloprovincialis*), against the human herpesvirus HSV-1 and HSV-2 by using Vero cells as a surrogate model [105]. According to the results, Myt-Tat treatment has significantly reduced the virus-induced cytopathic effects against both HSV-1 and HSV-2 viruses with a mean effective concentration (EC50) of 3.97 ± 0.94 µM (24.35 ± 5.74 µg/mL) against HSV-1(R2 = 0.9871) and 3.09 ± 1,02 µM (18.98 ± 6.27 µg/mL) against HSV-2(R2 = 0.9688). With increasing the concentration of the peptide, the number of the virus recovered after the treatment was decreased, and Myt-Tat treatment at 8.15 µM and above resulted in no virus recovery. These results indicate the therapeutic potential of Myt-Tat derived from mollusks. A study conducted by Defer and colleagues in 2009 [106] reported that the acidic extracts from *Cerastoderma edule*, *Ruditapes philippinarum*, *Ostrea edulis*, *Crepidula fornicate*, and *Buccinum undatum* showed both antibacterial and antiviral activity against human pathogens. They reported that the acidic extracts inhibited the Herpes simplex virus type 1 on Vero cell model. Moreover, it was proven that *C. edule* was most efficient in antiviral activity.

Economically important marine bivalve green mussel (*Perna viridis*), oyster *Crassostrea madrasensis*, *C. gryphoides*, and clam *Meretrix casta* were reported to possess high antiviral activity against influenza virus strain type A. By infecting fragments of ChAM from chicken embryos, the bivalve extracts’ viral inhibitory efficacy against human influenza viruses was assessed. According to the results, the extract made from *P. viridis* had the maximum difference for in vitro tests with influenza virus type-A (2.50 lg), and virus type B showed the greatest variation in the EID50 value (3.00 lg). The extracts of *V. cyprinoides* and *P. erosa* with influenza virus type A showed the greatest difference in EID50 values for in vivo tests (4.00 lg and 4.00 lg, respectively) [107].

The majority of described extracts from bivalve species with in vitro antiviral activity were compiled in a thorough study by Dang et al. in 2015 [108]. As reported, the viral attachment and transcription processes were directly inhibited by the mytilin and defensin from *M. galloprovincialis*. Defensins are 18–45 amino acid-long peptides with high cysteine content that are generated by the innate immune system and are very effective against a wide range of bacteria, fungi, and enveloped and non-enveloped viruses. A new DNase-like chemical that can prevent viral spread from the green-lipped mussel *P. viridis* was described by Muhammed Zafar Iqbal and Khan in 2016 [109]. They have investigated the DNAes, like bioactivity, for the naturally occurring non-proteinaceous compounds. These findings suggested the possibility of a source of AD against DNA Group I viruses. Khan and Muhammed Zafar Iqbal hypothesized that the sea mussels have developed certain defenses against viral infections that require more investigation.

**Figure 2:**
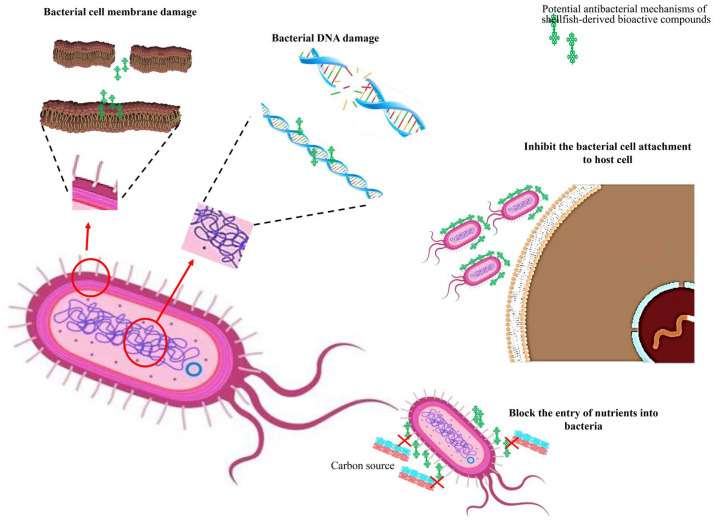
Potential antibacterial mechanisms of shellfish-derived bioactive compounds.

Figure 2 summarizes the potential antibacterial mechanisms of shellfish bioactive compounds.

## 6. Conclusion and Future Directions

In conclusion, this review highlights the anticancer and antimicrobial potential of shellfish-derived bioactive compounds, including peptides, lipids, polysaccharides, and marine-inspired derivatives. Current evidence demonstrates that these compounds can inhibit cancer cell proliferation and suppress microbial growth, with several studies reporting favorable biocompatibility and low toxicity in both in vitro and selected in vivo models. Collectively, these findings support the view that shellfish represent a valuable and underexplored source of bioactive molecules with relevance to human health.

However, despite this promise, the field remains at an early stage of development. Future research should prioritize the systematic isolation and structural characterization of active compounds from crude extracts to enable reproducibility and mechanistic clarity. In parallel, structure–activity relationship (SAR) studies are needed to identify key molecular features responsible for bioactivity and to guide compound optimization.

To strengthen translational relevance, validation in appropriate in vivo disease models should be expanded, particularly for compounds that have demonstrated robust in vitro efficacy. Rigorous assessment of toxicity, bioavailability, and pharmacokinetics will be essential to determine their suitability for therapeutic or nutraceutical development. In addition, comparative studies evaluating shellfish-derived compounds against existing anticancer and antimicrobial agents would provide critical benchmarks for efficacy and safety.

Finally, future investigations should explore innovative formulation and delivery strategies, including nanoparticle-based and antibody–drug conjugate approaches, to improve stability, targeting, and therapeutic index. Standardization of extraction methods and bioassay protocols across studies will further enhance cross-study comparability and accelerate progress in this field.

Overall, advancing shellfish-derived bioactives from discovery toward application will require an integrated strategy combining chemical characterization, mechanistic insight, in vivo validation, and translational development. With continued focused research, shellfish-derived compounds have strong potential to contribute to future anticancer and antimicrobial therapies.

## Figures and Tables

**Table 1:** Shellfish-derived bioactive compounds.

Bioactive Component	Source	Activity	Compound	Reference
Peptides	*Ruditapes philippinarum*	Antitumor	Taurine	[22]
	*Crenomytilus gryanus*	Antifungal, anti-HIV	Lectin	
	*Fissurella latimarginata*	Anticancer	Lectin	[23]
	*Mytilus galloprovincialis*	Anti-HIV 1	Defensin and Defensin like MGD1 &2	[24,25]
	*Meretrix meretrix*	Anti-lung cancer	Mere 15	[11,26]
	*Mytilus eduli*	Antimicrobial	Defensin	[27,28]
	*Ruditapes decussatus*	Antibacterial activity	Myticin 1, 2 and 3 9	[29]
	*Mytilus edulis chilensis*	Antimicrobial activity	Mytimycin	[30,31]
	*Hyas araneus*	Antimicrobial activity	Arasin 1&21	[32]
	*Carcinus maenas*	Antibacterial activity	Crustins	[33]
	*Scylla serrata*	Antibacterial activity	Scygonadin	[34]
	*Litopenaeus vannamei*	Antimicrobial activity	Penaeidins	[35]
	*Mytilus edulis*	Antimicrobial, antifungal, antiviral	Polyphemusin	[36,37]
	*Dolabella auricularia*	Antifungal, anticancer	Dolastatin	[38]
	*Mytilus galloprovincialis*	Antimicrobial	Mytilin	[32]
	*Elysia rufescens*, *Spisula polynyma*	Anti-microbial	Kahalalide F	[39]
	*Penaeus vanname*, *Penaeus setiferus*	Antimicrobial, antifungal	Penaeidin	[40]
	*Penaeus vannamei*, *Penaeus stylirostris*	Antifungal		[41]
	*Pleurobranchus forskalii*	Anti-tumor	Keenamide A	[39]
	*Fenneropenaeus chinensis*	Antimicrobial	Crustin	[42]
Fatty acids	*Crassostrea gigas*	Anticancer	docosahexaenoic acid	[43]
	*Crab & shrimp*	Anticancer	Eicosapentanic acid (EPA)	[44]
	*Hyas aureus*	Antibacterial activity	lysoglycerolipids/glycerides	[45]
	*Thalamita crenata*	Anticancer	Omega 3-fatty acid	[46]
Polysaccharides	*Hemicentrotus pulcherrimus*	Anticancer	HPP-1S	[47]
	*Penaeus vannamei*	Anticancer	chondroitin sulfate	[48]
	*Donax variabilis*	Anticancer	Polysaccharides	[49]

**Table 2:** Marine mollusk-derived anticancer drugs.

Compound/Drug	Molecular Target	Cancer Type	Marine Linkage	Reference
Enapotamab vedotin	AXL-RTK/Nactin-4	Ovarian cancer, cervical cancer, eEndometrial cancer, advanced or metastatic solid tumours	ADC containing dolastatin-derived payload; marine-inspired	[78,79]
CX-2029	Tissue factor	Solid tumour, head and neck cancer, non-small-cell lung cancer, pancreatic cancer, diffuse large B-cell lymphoma	Marine natural product–inspired ADC	[80,81]
RC48	HER2	Urothelial carcinoma, advanced cancer, gastric cancer, HER2-overexpressing gastric carcinoma, advanced breast cancer, solid tumours	Synthetic analogue of dolastatin-10, a cytotoxic peptide originally isolated from the marine sea hare *Dolabella auricularia*	[82,83]
Telisotuzumab vedotin	c-Met	Solid tumours	Dolastatin analogue (MMAE); marine natural product derivative	[84,85]
Ladiratuzumab vedotin	LIV-1 and microtubules	Breast cancer	Marine-derived auristatin payload; clinical-stage ADC	[86,87,88]
AGS-16C3F	ENPP3 and microtubules	Renal cell carcinoma	ADC incorporating auristatin (MMAF) payload derived from marine mollusk dolastatin-10	[89,90]
W0101	IGF-R1	Advanced or metastatic solid tumours		[78]
ARX-788	HER2 and microtubules	Breast cancer, gastric cancer	Auristatin-based payload; marine-inspired cytotoxin	[91,92]
XMT-1536	NaPi2b and microtubules	Solid tumours	ADC using auristatin (dolastatin-derived) payload originally sourced from marine mollusk *Dolabella auricularia*	[93,94]

## Data Availability

The original contributions presented in this study are included in the article. Further inquiries can be directed to the corresponding authors.
